# Upper extremity nerve block: how can benefit, duration, and safety be improved? An update

**DOI:** 10.12688/f1000research.7292.1

**Published:** 2016-05-18

**Authors:** Metha Brattwall, Pether Jildenstål, Margareta Warrén Stomberg, Jan G. Jakobsson

**Affiliations:** 1 Department of Anaesthesiology and Intensive Care, Unit of Day Surgery, Sahlgrenska University Hospital, Göteborg, Sweden; 2 Institute of Health and Care Sciences, The Sahlgrenska Academy, University of Gothenburg, Gothenburg, Sweden; 3 Department of Anaesthesiology and Intensive Care, Örebro University Hospital, Örebro, Sweden; 4 Department of Anaesthesia & Intensive Care, Institution for Clinical Science, Karolinska Institute, Danderyds University Hospital, Stockholm, Sweden

**Keywords:** upper extremity block, peripheral blocks, ultra-sound guided, anaesthesia, interscalene, supraclavicular, infraclavicular, axillary plexus

## Abstract

Upper extremity blocks are useful as both sole anaesthesia and/or a supplement to general anaesthesia and they further provide effective postoperative analgesia, reducing the need for opioid analgesics. There is without doubt a renewed interest among anaesthesiologists in the interscalene, supraclavicular, infraclavicular, and axillary plexus blocks with the increasing use of ultrasound guidance. The ultrasound-guided technique visualising the needle tip and solution injected reduces the risk of side effects, accidental intravascular injection, and possibly also trauma to surrounding tissues. The ultrasound technique has also reduced the volume needed in order to gain effective block. Still, single-shot plexus block, although it produces effective anaesthesia, has a limited duration of postoperative analgesia and a number of adjuncts have been tested in order to prolong analgesia duration. The addition of steroids, midazolam, clonidine, dexmedetomidine, and buprenorphine has been studied, all being off-label when administered by perineural injection, and the potential neurotoxicity needs further study. The use of perineural catheters is an effective option to improve and prolong the postoperative analgesic effect.

Upper extremity plexus blocks have an obvious place as a sole anaesthetic technique or as a powerful complement to general anaesthesia, reducing the need for analgesics and hypnotics intraoperatively, and provide effective early postoperative pain relief. Continuous perineural infusion is an effective option to prolong the effects and improve postoperative quality.

## Introduction

Peripheral blocks have been part of anaesthetic techniques used for upper extremity surgery for decades. Intravenous regional anaesthesia, the so-called Bier block, was described in 1908 by A. G. Bier ^1^. The interscalene block was also initially described more than a century ago, in the early 1900s ^2^. The brachial plexus came some years later ^3^. These blocks were initially done by identifying anatomical landmarks and eliciting paraesthesia. The introduction of the nerve stimulator technique in the late 1980s made a marked change in practice: from being a technique used only by experienced individuals, it became far more commonly used ^4^. The introduction of the ultrasound technique during the last decade has further enhanced performance. A Cochrane review suggests that the ultrasound-guided block technique further improves the success and ease of performance ^5^. The availability of information via websites ^6^ and demo films ^7, 8^ has made the technique much appreciated, and it is now commonly used by younger colleagues. A recent paper by Sehmbi *et al*. describes the ultrasound-guided technique for various upper extremity blocks ^9^. Ultrasound guidance further increases the success rate and, when used in combination with nerve stimulation, it provides, as of today, the highest degree of safety and success ^10, 11^. A recent Cochrane systematic review supports the efficacy of the ultrasound-guided block technique but also addresses the importance of experience and the training curve of ultrasound vs. other techniques ^5^. In the March-April 2016 issue of *Regional Anesthesia and Pain Medicine*, Neal *et al*. ^12^ have also provided an executive summary of “Evidence-Based Medicine Assessment of Ultrasound-Guided Regional Anesthesia”. They concluded that there is high-level evidence supporting ultrasound guidance, contributing to superior characteristics with selected blocks, although absolute differences with the comparator technique, e.g. nerve stimulation, are often relatively small, especially for upper extremity blocks.

The upper extremity blocks may be divided into the following ^13^:

- Interscalene, shoulder surgery
- Supraclavicular, the entire arm
- Infraclavicular, the elbow and below
- Axillar plexus, from below the elbow

The aim of the present paper is to provide an update and overview of the clinical usage of upper extremity nerve blocks, how analgesic effect can be prolonged, and how safety can possibly be improved, strengthening the benefit vs. risk for their clinical use.

We conducted a public domain literature search using PubMed looking for papers addressing upper extremity block, ultrasound-guided block, and pain, with a focus on meta-analyses and reviews published in 2010 and onwards.

## Efficacy and outcome

### Interscalene block

Today, the interscalene block is well established for intraoperative as well as postoperative pain management associated with shoulder surgery. In May 2015, Abdallah *et al*. performed a meta-analysis ^14^, which showed a clear effect on pain for up to 6 hours during movement and 8 hours at rest and an opioid-sparing effect for up to 24 hours after surgery. Ullah *et al*. ^15^ searched for studies assessing continuous perineural interscalene block for pain relief after major shoulder surgery, but they were not able to conduct a meta-analysis because of the lack of studies. They still concluded that the catheter technique provides better pain relief than parenteral analgesics. There are further studies supporting the beneficial effects of the perineural catheter technique improving postoperative pain course. Fredrickson *et al*. ^16^ compared single-shot to continuous perineural infusion following minor shoulder surgery and found significant positive effects. Salviz *et al*. ^17^ likewise found superior postoperative analgesia up to day 7 when comparing the single-shot to the catheter technique. Patients in the continuous interscalene block group received 20 mL of 0.5% ropivacaine as a bolus through a catheter, whereas single-shot patients received the same injection volume through a needle. The continuous group of patients received a further infusion of 0.2% ropivacaine at 5 mL/hour with a patient-controlled bolus of 5 mL hourly for 48 hours. Mariano *et al*. ^18^ compared single-shot to a 48-hour continuous infusion with ropivacaine 0.2 mg/mL. They also found superior pain relief, improved sleep, and higher patient satisfaction. Indeed, perineural catheter techniques are an effective option.

### Supraclavicular plexus block

Supraclavicular block carries the risk of pneumothorax and also the development of transient Horner’s syndrome. However, the ultrasound-guided technique has facilitated its performance, and there is a growing interest in the block. In 2014, Sadowski *et al*. published a comprehensive review on its renascence following the introduction of the ultrasound-guided technique ^19^. Gamo *et al*. presented their experience with the ultrasound-guided supraclavicular block technique in 202 patients ^20^. They showed the block had a rapid procedure time (average 4 minutes), good intraoperative conditions, a mean surgery time of 75.2 minutes with a range of 6 to 232, and a mean of 437 minutes (range 171 to 992) of postoperative analgesia. Transient Horner’s syndrome was observed in 10% of patients. Vaghadia *et al*. compared ropivacaine and bupivacaine for supraclavicular plexus block performed by paraesthesia or nerve stimulation technique in 104 ASA physical status 1–3 patients scheduled for upper arm surgery. Long and effective anaesthesia/analgesia was achieved with ropivacaine 7.5 mg/mL similar to 5 mg/mL bupivacaine without differences between the two groups. The mean duration of analgesia, time to need for rescue analgesia, was 11–12 hours ^21^.

### The infraclavicular plexus block

There is one recent meta-analysis assessing the available evidence on the infraclavicular block technique for perioperative use. In 2013, Chin *et al*. published a Cochrane systematic review on the use of infraclavicular plexus block for surgery of the lower part of the arm ^22^. They concluded, based on the 15 studies included, that infraclavicular plexus block is an effective alternative to supraclavicular and axillary block, providing superior intraoperative tourniquet pain control as compared to single injection axillary block and faster performance as compared to multi-injection axillary block. It had a similar postoperative analgesic duration as compared to other peripheral blocks (supraclavicular and axillary). Overall, it seems to be an advantageous technique over the traditional axillary block.

### The axillary plexus block

Chin *et al*. also conducted a Cochrane systematic review on axillary plexus block, assessing single, double, and multiple injection techniques ^23^. In all, 21 trials were included, presenting results from a total of 2148 participants who received regional anaesthesia for hand, wrist, forearm, or elbow surgery. Studies with trans-arterial and nerve stimulator techniques were included. The multiple injection technique was found to improve success rates but demonstrated adequate surgical anaesthesia and motor block as compared to the single injection technique. No significant difference was found in analgesia failure, complications, and patient discomfort. However, the time for block performance was significantly shorter for single and double injection techniques as compared with multiple injections. There are two recent papers comparing nerve stimulation and ultrasound guidance for axillary plexus block. Kumar *et al*. found both techniques to be equally safe and effective ^24^. Meierhofer *et al*. found similar results ^25^. No major difference in success rate between nerve stimulation and ultrasound technique was found; however, the authors commented that the skills required for each respective technique must be taken into account.

## Risk and side effects

The upper extremity block may cause side effects, such as nerve damage, intravascular injection causing local anaesthesia toxicity, diaphragm dysfunction, and pneumothorax. There is a recent update from the American Society of Regional Anesthesia and Pain Medicine by Neal ^26^: this analysis concluded that ultrasound guidance has no significant effect on the incidence of postoperative neurologic symptoms. The ultrasound-guided block technique reduces the incidence and intensity of hemidiaphragmatic paresis but in an unpredictable manner. Ultrasound guidance reduces the risk of local anaesthesia toxicity and may also reduce the predicted frequency of pneumothorax, but this requires training in visualisation of the needle ^27^. Also, with regard to the volume and concentration, the lower dosage needed has an impact with less of an effect on the diaphragm ^28, 29^. Thackeray *et al*. ^30^ showed that an ultrasound-guided interscalene brachial plexus catheter placement with 20 mL of 0.125% bupivacaine caused significantly less diaphragm dysfunction as compared to 0.25% bupivacaine. Stundner *et al*. ^31^ conducted an elegant study, administering patients with ropivacaine 0.75%, either 20 or 5 mL, plus contrast dye followed by magnetic resonance imaging. Both groups experienced fast onset and adequate intraoperative and postoperative analgesia, with no significant differences in pain scores. The spread was more pronounced with the higher volume, diaphragm dysfunction occurred twice as frequently, and changes from baseline peak respiratory flow rate were in the 20 mL group. Horner’s syndrome may also be associated with upper extremity plexus blocks. Tran *et al*. ^32^ compared ultrasound-guided supraclavicular, infraclavicular, and axillary brachial plexus blocks for upper extremity surgery of the elbow, forearm, wrist, and hand. They found all three blocks to be effective; however, the axillary block required longer time to perform and the supraclavicular block was associated with a higher incidence of Horner’s syndrome (37.5% vs. 0−5%; both P <0.001).

## Techniques in order to facilitate block quality and duration

The single-shot upper extremity blocks have effective anaesthesia duration of hours when performed with a long-lasting local anaesthetic (bupivacaine, levobupivacaine, or ropivacaine). The analgesic effect wears off within the duration for a long-lasting local anaesthetic (6–10 hours). Abdallah *et al*. ^14^ raised the question of whether the benefit of the early pain relief may be overwhelmed by the pain “rebound” when the block wears off. Pain during the first postoperative evening and night following discharge does cause concern. Sunderland *et al*. found that patients who had a block required more unplanned healthcare visits as compared to patients who had general anaesthesia, mostly due to pain ^33^. There is without doubt an interest in prolonging the analgesic effect, reducing postoperative pain, and this can be done by the addition/combination of different adjuncts. There are several techniques used in order to improve quality and extend the duration of postoperative analgesia, and different adjuncts have been tested in order to facilitate the quality and the duration of peripheral nerve blocks. It must, however, be acknowledged that the use of adjuncts for perineural administration is off-label and potential nerve toxicity needs further study. Adding active medication perineurally must be done with the benefit and potential risk taken into account for each individual patient. Williams *et al*. ^34^ conducted an *in vitro* study assessing the neurotoxicity from ropivacaine sole agent and in combination with clonidine, buprenorphine, dexamethasone, and midazolam and showed clear signs of neurotoxicity, suggesting that further studies are warranted in order to elucidate risk. Kirksey *et al*. published a systematic review in September 2015 on adjuncts for improving peripheral blocks, and they concluded that the addition of perineural buprenorphine, clonidine, dexamethasone, dexmedetomidine, or magnesium showed a consistently prolonged duration of peripheral nerve block ^35^. The benefit vs. risk associated with the co-administration of drugs perineurally must, however, be acknowledged. Bailard *et al*. addressed the benefit vs. risk and commented that there are an increasing number of studies suggesting that systemic administration may provide more or less similar effects to perineural administration and possibly a decreased risk, at least for nerve toxicity ^36^.

## Clonidine

Alpha-2-agonists have been added to upper extremity blocks for decades. In 1996, Singelyn *et al*. conducted a dose-finding study assessing the minimal effective clonidine dose able to improve axillary block ^37^. Axillary brachial plexus block was performed with nerve stimulator and 40 mL 1% mepivacaine plus 1:200,000 epinephrine local anaesthesia doses. The control group received no clonidine. In the other groups, increasing doses of clonidine (0.1, 0.2, 0.3, 0.4, 0.5, 1, and 1.5 mg/kg) were added to the local anaesthetic solution. Both anaesthesia and analgesia duration were increased, with the lowest effective dose being 0.5 mg/kg clonidine, with no clear additional effect in further increasing the dose. The anaesthesia duration was increased from 260 minutes up to 310 at 0.5 mg/kg, where the effect plateaued, and the analgesia showed a small dose response from 260 minutes up to 490 minutes, with only limited additional effects from 1.0 and 1.5 mg/kg. The linear trend in the duration of analgesia corresponding to doses of 0.5, 1, and 1.5 pg/kg was not significant (P = 0.97), indicating no further increase in duration with dose. No effect on the onset or quality of block was noticed. McCartney *et al*. performed the first meta-analysis on the effects of adding clonidine to peripheral blocks ^38^. In all, 1385 patients in 27 studies were included, and five studies included a systemic control group. The dose of clonidine studied ranged from 30 to 300 mg. There were 15 studies that supported the use of clonidine as an adjunct to peripheral nerve block, with 12 studies failing to show any benefit. Clonidine appeared to prolong analgesia when added to intermediate-acting local anaesthetics for axillary and peribulbar blocks. In 2009, Pöpping *et al*. conducted a meta-analysis of the available evidence on the addition of clonidine to peripheral blocks and concluded that there was still a lack of clear evidence of dose responsiveness for beneficial as well as for harmful effects ^39^. The combination of clonidine and intermediate or long-acting local anaesthetics for single-shot peripheral nerve or plexus blocks prolongs the duration of analgesia and motor block by about 2 hours. They also commented on the potential risk of alpha-2-agonist-associated side effects; therefore, increased risk of hypotension, fainting, and sedation may limit its usefulness. Furthermore, the optimal dose, the dose responsiveness to balance benefit and duration, and the side effects remain unclear.

## Dexmedetomidine

Swami *et al*. conducted a randomised double blind study in 60 patients where the same dosage of clonidine or dexmedetomidine as an adjuvant to bupivacaine in supraclavicular brachial plexus block was compared ^40^. There was no statistically significant difference in onset of sensory and motor block between the two groups. They found in this small study, however, that dexmedetomidine provided superior postoperative effects as compared to clonidine. The duration of sensory block and motor block was mean 227 ± 48 and 292 ± 59 minutes, respectively, in the clonidine group of patients and mean 414 ± 87 and 472 ± 90 minutes, respectively, in the dexmedetomidine group. The duration of analgesia (time to requirement of rescue analgesia) in the dexmedetomidine group was 456 ± 97 minutes, while in the clonidine group it was 289 ± 62 minutes. Statistically, this difference was significant (P=0.001). The number of patients achieving what was considered an excellent block was also higher in the dexmedetomidine group of patients (80%) as compared with the clonidine group (40%) (P <0.05). In 2013, Abdulla *et al*. conducted a meta-analysis of studies assessing the co-administration of dexmedetomidine to perineural blocks ^41^. Four out of the nine studies included were studies of brachial plexus blocks. Dexmedetomidine significantly increased analgesia duration for spinal block, but the increase was not significant for the plexus block studies. The duration on motor function and time to first analgesic request were prolonged for both intrathecal and brachial plexus block. The authors concluded that there are still insufficient safety data to support perineural dexmedetomidine use in the clinical setting. Wu *et al*. performed a second meta-analysis, which was published in early 2014 ^42^. In all, 1092 patients from 16 randomised controlled clinical trials were included in the analysis. They found, likewise, that neuraxial dexmedetomidine improved postoperative analgesia, with a mean difference of almost 7 hours, and significantly decreased postoperative pain intensity. Dexmedetomidine co-administration, however, increased the risk of bradycardia, with an odds ratio of 2.68.

The most recent study from September 2015 by Kaur *et al*. ^43^ assessing the effects of dexmedetomidine for plexus block also found a statistical difference between the groups in motor blockade and postoperative pain with an advantage seen in the group of patients who received dexmedetomidine. They also noticed some minor haemodynamic side effects from dexmedetomidine, such as bradycardia in two patients; however, none of these patients needed any treatment.

## Dexamethasone

In 2014, Choi *et al*. conducted a systematic review and meta-analysis of randomised trials comparing brachial plexus block performed with local anaesthetic with or without additional perineural dexamethasone ^44^. In all, 801 patients from nine trials were included, with 393 patients receiving dexamethasone (4–10 mg). The meta-analysis showed that dexamethasone prolonged the analgesic duration for long-acting local anaesthetics from 730 to 1306 minutes, with a mean difference of 576 minutes, and for intermediate local anaesthetics from 168 to 343 minutes, with a mean difference of over 175 minutes. The motor block effect was also prolonged from 664 to 1102 minutes. An important finding was that the most recent trial demonstrated equivalent prolongation with perineural or systemic administration of dexamethasone compared with placebo. Also in 2014, de Oliveira Jr *et al*. ^45^ published a review article regarding perineural dexamethasone as an adjunct to the brachial plexus blocks. In all, 760 subjects from nine randomised trials with dexamethasone were included. They found a clear positive effect of perineural dexamethasone over control for analgesia, a mean prolongation of 473 (264 to 682) minutes, and a motor blockade duration of 500 (154 to 846) minutes. Postoperative opioid consumption was also reduced in the perineural dexamethasone group compared to control (-8.5 [-12.3 to -4.6] mg of intravenous morphine equivalents). In 2015, Albrecht *et al*. published a systematic review and meta-analysis regarding the safety and efficacy of perineural dexamethasone as an adjunct for peripheral nerve block ^46^. They found, likewise, a prolongation of postoperative analgesia. Dexamethasone increased the mean duration of analgesia by 233 minutes when combined with a short- or medium-term action local anaesthetic and by 488 (419–557) minutes when injected together with long-term action local anaesthetics (p <0.00001 for both). They could not see any clear dose response, the prolongation was not significantly dependent on the dose (4 to 10 mg dexamethasone), and it seems that intravenous and perineural administration has an equivalent effect. A fourth meta-analysis by Huynh *et al*. was recently published, in November 2015 ^47^. In all, 1054 patients, 512 receiving perineural dexamethasone, from 12 trials were included. Ten were assessing the effects for brachial plexus nerve block. Doses of between 4 and 10 mg dexamethasone-containing local anaesthetic solutions showed a faster onset of action and resulted in a significant prolongation in the duration of analgesia (mean difference 351 minutes [P < 0.001] and motor blockade mean 277 minutes [P < 0.001]) compared with sole local anaesthetic. Time to onset of sensory and motor blocks was also significantly reduced with dexamethasone by about 1 minute. Dexamethasone significantly decreased postoperative nausea and vomiting by 9 vs. 27%. Thus, dexamethasone approximately doubled the duration of postoperative analgesia when it was combined with intermediate-acting (lidocaine and mepivacaine) or long-acting (bupivacaine and ropivacaine) local anaesthetics and had a clinically important effect in reducing postoperative nausea and vomiting.

### Opioids

There are sparse data around the addition/co-administration of buprenorphine for upper extremity blocks. In 2012, Behr *et al*. published a study assessing the perineural vs. systemic effects of buprenorphine in patients scheduled for shoulder arthroscopic surgery for a rotator cuff tear under middle interscalene brachial plexus block with 29.5 mL of 0.75% levobupivacaine ^48^. The patients were randomised to receive additionally either saline or intramuscular buprenorphine 0.15 mg or epineural buprenorphine 0.15 mg. They found that the duration of both sensory block and postoperative analgesia was longer (P <0.05) in patients who had received epineural buprenorphine (856.1 ± 215.2 and 1049.7 ± 242.2 minutes) than in patients who had received intramuscular buprenorphine (693.6 ± 143.4 and 820.3 ± 335.3 minutes) or saline (488.3 ± 137.6 and 637.5 ± 72.1 minutes). The need for postoperative rescue analgesics was also lower in the epineural buprenorphine group than in the other two groups. Buprenorphine has also gained huge interest, and a recent paper by Kosel *et al*. suggested it is a “unique opioid adjuvant in regional anesthesia” ^49^. Thus, available clinical data suggest that local anaesthesia combined with clonidine, buprenorphine, or dexamethasone has beneficial effects prolonging the analgesic effect and reducing pain and need for rescue medication and do not appear to alter local anaesthetic neurotoxicity ^50^. There are, however, *in vitro* studies raising concern around neurotoxicity both by ropivacaine plain and when combined with midazolam and dexamethasone ^34^. It should, however, be recalled that perineural administration of these compounds is, in most countries, still considered to be “off-label use”.

## Perineural catheters

An alternative option is to use an indwelling perineural catheter for continuous administration of local anaesthetic with or without adjuncts. Ullah *et al*. published a paper aimed at the performance of a meta-analysis of available studies assessing the continuous technique ^15^. However, they were unable to make any analysis because of too few papers and heterogeneous design. They still commented that the continuous interscalene block seems to provide better pain relief as compared to parenteral opioids and to have a favourable safety profile when ultrasound technique was used. The improved safety using ultrasound-guided block technique and subsequent lower volume of local anaesthesia has been documented repeatedly ^51, 52^. It also facilitates success rate and shortens time to onset ^53^. Ilfeld *et al*. have published two papers supporting the effective pain management with continuous perineural catheter in ambulatory shoulder surgery, showing significant effect on pain and discharge time ^54, 55^.

## Future perspectives

Upper extremity blocks provide effective analgesia for the duration of the local anaesthetic used. They provide an opioid-sparing effect during the early postoperative course up to 24 hours after surgery; however, the more protracted/long-term benefits are not extensively studied. The addition of adjuncts facilitates the duration and possibly reduces the risk of pain rebound when the analgesic effects wear off. It seems of importance to conduct further high-quality studies including more long-term recovery and outcomes ^56^. Studies that compare the different blocks based on combination of local anaesthesia adjuncts that facilitate and extend the analgesia duration are required. There is also a need for further studies on mechanism of action, assessing whether the additive effect of adjuncts is local or merely systemic. The studies should be three armed with sole local anaesthesia, perineural adjunct/placebo systemic, and placebo perineural and adjunct systemic. Assessing not only the early first 24-hour postoperative pain effects but also the overall recovery and the effects for up to at least a week after surgery using validated recovery tools such as the Postoperative Quality of Recovery scale ^57^ are warranted. Fischer and Bosch did indeed address this in a recent paper ( *Does regional anaesthesia improve outcome after surgery?*), ** questioning the ** explicit benefits of the peripheral blocks unless combined with a dedicated enhanced recovery pathway program ^58^.

## Summary and conclusion

The ultrasound technique has had a major impact on anaesthesiologists’ interest in performing upper extremity blocks. The increased interest in, training in, and use of the ultrasound technique have also reasonably improved the safety of the performance of the upper extremity, interscalene plexus, supraclavicular/infraclavicular as well as axillary plexus blocks. An update found evidence that upper extremity blocks provide effective intraoperative and early postoperative analgesia, reducing opioid consumption in the first 24 hours, but may cause rebound pain ^59, 60^. The addition of dexamethasone and/or alpha-2-agonist may improve the quality of and extend the duration of analgesia. However, further studies are warranted; assessing whether adjuncts have a local action or can be administered systemically and further assessing the effects of the block technique not only on early pain but also on the quality of recovery during the early and more protracted/long-term effects, beyond the first 24 to 48 hours, is warranted. The continuous perineural infusion is a feasible alternative, providing effective analgesia and improving quality of recovery, sleep quality, and patient satisfaction.

## Key message

- Upper extremity surgery can be performed safely in peripheral block as sole anaesthesia or in combination with sedation and general anaesthesia as needed.
- Planning and proper logistics are of importance when implementing regional anaesthesia.
- Training and skill in the regional anaesthesia technique must be secured.
- Factors of importance include time to onset of block, duration of surgical anaesthesia, and duration of postoperative analgesia.
- The use of nerve stimulation or even more ultrasound guidance improves the success rate of the block and reduces the risk of side effects.
- Use of long-acting local anaesthetic solution delays onset but prolongs duration of effective anaesthesia.
- Addition of adjuncts prolongs the duration of postoperative analgesia, but further studies are warranted in order to better document safety and whether systemic administration is equally effective as compared to perineural administration.
- Perineural catheters and continuous infusion are also an effective alternative to prolong the analgesic effects.
